# Anti-Corrosion Properties of Tantalum-Based Composite Films Prepared by Atomic Layer Deposition

**DOI:** 10.3390/nano16110688

**Published:** 2026-06-01

**Authors:** Ge Xu, Wei Yu, Minxuan Zhang, Fei Cai, Qiushun Zou, Jianheng Li, Jing Hu, Zhixin Wan, Shihong Zhang

**Affiliations:** 1 Key Laboratory of Green Fabrication and Surface Technology of Advanced Metal Materials (Ministry of Education), Anhui University of Technology, Ma’anshan 243002, China; 15971965339@163.com (G.X.); yw220794@163.com (W.Y.); zmx15155366680@163.com (M.Z.); caifei20150126@163.com (F.C.); 2 Zhejiang Key Laboratory of Advanced Optical Functional Materials and Engineering Research Center for Advanced Infrared Photoelectric Materials of Zhejiang Province, Ningbo University, Ningbo 315211, China; zouqiushun@nbu.edu.cn; 3 Anhui ADChem Semiconductor Technology Company Limited by Shares, Hefei 231200, China

**Keywords:** atomic layer deposition, Ta-based thin films, tantalum oxynitride, multilayer films, corrosion resistance performance

## Abstract

Reported herein is tantalum (Ta)-based film, including TaN, TaO_x_, composite TaO_x_N_γ_, multilayered TaN/TaO_x_-(5:5) and TaN/TaO_x_-(10:10), prepared by atomic layer deposition (ALD) technology via adjusting the sub-cycle of TaN and TaO_x_ films. The influence of different growth parameters on microstructure, crystal form, chemical bonding state and corrosion resistance of Ta-based films was systematically investigated. Representative results include the following: (1) The surface of the Ta-based films prepared by ALD is continuous, dense and smooth, and the root mean square roughness (*R*q) of those are TaN: 0.74 nm, TaO_x_: 0.69 nm, TaO_x_N_γ_: 0.55 nm, TaN/TaO_x_-5:5: 0.56 nm and TaN/TaO_x_-10:10: 0.77 nm. (2) The TaN film presents a polycrystalline structure with good crystallinity, while the incorporation of oxygen significantly inhibits the crystallinity of the film. (3) Electrochemical tests in 3.5 wt.% NaCl solution and neutral salt spray experiments show that ALD deposition of Ta-based films can significantly improve the corrosion resistance of carbon steel substrates. The order of corrosion resistance of different films is TaO_x_N_γ_ film > TaN/TaO_x_ multilayer film > TaN film. Among them, the TaO_x_N_γ_ film exhibited the most excellent corrosion resistance, with a charge transfer resistance (*R*_ct_) as high as 24.75 Ω·cm^2^ and a corrosion current density (*I*_corr_) as low as 1.20 × 10^−6^ A/cm^2^, and no obvious rusting phenomenon was observed on the surface of that film after the 2 h neutral salt spray test.

## 1. Introduction

The corrosion failure issues of marine engineering equipment, precision electronic components, and new energy infrastructure in harsh operating environments, particularly in marine atmospheres containing high concentrations of chloride ions (Cl^−^) or in saline water media, have become increasingly significant [1,2,3,4]. Cl^−^ exhibits strong corrosivity toward metallic materials, capable of rapidly degrading the passive film and initiating localized corrosion phenomena such as pitting and crevice corrosion, thereby severely compromising the reliability and service life of equipment and components [5]. Tantalum (Ta)-based films, particularly tantalum nitride (TaN) and oxygen–nitrogen co-doped tantalum (TaO_x_N_γ_), have emerged as promising candidates for high-performance protective coatings due to their exceptional chemical inertness, high hardness, and low chloride ion (Cl^−^) diffusion coefficient [6,7,8]. In oxygen-containing environments, the TaN film naturally develops a thin, stable TaO_x_ passivation layer on its surface, which exhibits extremely low ionic conductivity and thereby effectively suppresses anodic dissolution of the underlying metal substrate [9]. In contrast, the TaO_x_N_γ_ film demonstrates enhanced protective capability. When localized damage occurs, environmental water and oxygen can react with the exposed TaO_x_N_γ_ to form a protective TaO_x_ layer, which seals the defect and restores the coating’s integrity [10]. Research indicates that, compared to pure Ta films, both TaN and TaO_x_N_γ_ significantly shift the pitting potential of low-carbon steel or stainless steel toward more noble values in simulated seawater or salt spray environments, while reducing the corrosion current density by one to three orders of magnitude, highlighting their superior corrosion protection performance. Hirpara et al. [11,12] used radio frequency reactive magnetron sputtering to deposit Ta_2_O_5_, Ta_3_N_5_, and TaON coatings on 304 stainless steels, systematically investigating the influence of coating composition and surface morphology on corrosion resistance. The TaON coating exhibited hydrophobic properties and nanoscale roughness in 1 M NaCl solution, with a corrosion current density as low as 10^−11^ A/cm^2^. Additionally, they studied the corrosion resistance of TaO_x_N_γ_ coatings in dilute hydrochloric acid and found that the corrosion current density was effectively reduced by 60–80%. Their studies confirmed that tantalum oxynitrides are highly promising corrosion protection materials. However, to the best of our knowledge, no systematic study has been reported on atomic layer deposition (ALD) homogeneous TaO_x_N_γ_ films and TaN/TaO_x_ multilayers for corrosion protection of steel substrates. The ALD route offers unique advantages, including sub-nanometer thickness control, exceptional conformality on complex geometries (e.g., deep holes or precision components), and ultra-low porosity [13]. The resulting highly compact structure effectively blocks the penetration of moisture, oxygen, and corrosive ions (e.g., Cl^−^), while the minimal porosity ensures the continuity and integrity of the protective barrier, thereby suppressing localized pitting corrosion and enhancing the long-term reliability of the underlying substrate [14]. Furthermore, ALD proceeds via sequential, self-limiting surface reactions, forming ultra-thin, conformal interfaces with strong chemical bonding. This leads to excellent film adhesion, which is critical for resisting environmental degradation and delamination [15]. Previous studies have demonstrated the efficacy of ALD in surface protection applications. For example, C. X. Shan (2008) [16] significantly improved the corrosion resistance of stainless steel in a 3 wt.% NaCl solution by depositing a 50 nm amorphous TiO_2_ coating, resulting in a positive shift of the corrosion potential by 0.33 V, a 90% reduction in current density, and a tenfold increase in impedance. Similarly, Belén Díaz (2011) [17] reported that Al_2_O_3_ coatings with a thickness of 10 nm or more enhanced the surface performance of 100Cr6 carbon steel in a 0.2 M NaCl environment. Furthermore, research has indicated that multilayer structures can further optimize protective performance. For instance, E. Marin (2012) [18] demonstrated that a TiO_2_/Al_2_O_3_ multilayer coating exhibited superior corrosion resistance on AISI 316L stainless steel compared to its single-layer counterpart. Belén Díaz et al. (2014) [19] also found that the Al_2_O_3_/TaO_x_ nano-multilayer films exhibited higher stability than the single-layer films in an acidic NaCl environment (pH = 2). In addition, Jarmo Leppäniemi et al. [20] employed ALD to fabricate Al_2_O_3_/TiO_2_ nanolaminates, successfully sealing pinhole defects in PVD CrN coatings and achieving a two-order-of-magnitude reduction in corrosion current density. It provides complementary evidence for the unique advantages of ALD multilayer films in enhancing coating density and blocking corrosive species diffusion pathways. Unlike PVD coatings (which are line-of-sight, columnar, and prone to pinholes), ALD provides conformal coverage on complex geometries, near-zero porosity, atomic-scale multilayer mixing, and strong chemisorbed interfaces. These unique features directly account for the superior corrosion resistance of our ALD-derived films. To date, most published studies have focused on ALD-grown single-layer Al_2_O_3_ and TiO_2_ films and their multilayer architectures. No literature has systematically prepared and compared the corrosion protection properties of homogeneous TaO_x_N_γ_ amorphous solid solution films, TaN/TaO_x_ nanoscale multilayers, and pure TaN and TaO_x_ single-layer films using the ALD technique on the same platform.

Based on the aforementioned background and the necessity of expanding the material system, this study employs ALD to fabricate a series of tantalum-based composite films and systematically evaluate their corrosion protection performance, thereby expanding the application of ALD protective films in metal corrosion protection and enriching the related material systems. It aims to overcome existing limitations by innovatively employing ALD technology to design and fabricate two types of composite protective systems: (1) Homogeneous TaO_x_N_γ_ films: By utilizing a 1:1 cyclic ratio for the alternating growth of TaN and TaO_x_ sub-cycles, precise compositional control at the atomic scale is achieved to construct uniform composite films. (2) Multilayered TaN/TaO_x_ films: Alternating deposition of TaN and TaO_x_ sub-layers with precisely controlled individual layer thicknesses enables the construction of a strongly interface-coupled multilayered structure. The influence of ALD process parameters on the microstructure, crystalline phase, and chemical composition of the films was systematically investigated. These films were applied to protect AISI 1045 steel substrates, and their protective performance and underlying mechanisms were comprehensively evaluated in a simulated marine corrosion environment (3.5 wt.% NaCl solution, 25 °C). This evaluation included: assessing the corrosion rate and corrosion resistance via potentiodynamic polarization tests; analyzing interfacial charge transfer behavior using electrochemical impedance spectroscopy (EIS); combining standard salt spray testing to statistically examine the evolution of corrosion pits; and elucidating the corrosion inhibition mechanisms-oxygen-nitrogen co-passivation for the homogeneous film and interfacial barrier blocking for the multilayer film.

## 2. Experiment

### 2.1. Preparation of ALD-TaN Thin Films

The Ta-based films were grown in a commercial ALD system (Jiangsu MNT Micro Nanotech Co., Ltd., MNT-GPD200Oz-L2S3G4, Nanjing, Jiangsu, China) by using (t-butylimido)tris(diethylamino)tantalum (TBTDET, 6N purity) as metal precursor and high-purity ammonia (NH_3_, 99.999%, Nanjing Special Gas Company, Nanjing, Jiangsu, China) as the co-reactant. Single-crystal silicon wafers (Si (100), Zhejiang Lijing) and AISI 1045 carbon steel (composition in wt.%: C: 0.42~0.50, Si: 0.25, Mn: 0.71, *p*: <0.05, S: 0.01, Ni: 0.03, Cr: 0.03, Fe: balance) were employed as substrates. It should be noted that the Si substrates were employed for high-resolution microstructural and chemical analyses, whereas the AISI 1045 steel substrates were used for corrosion and salt spray testing. The Si substrate was ultrasonically cleaned in acetone and ethanol for 15 min each, then dried with high-purity N_2_. The AISI 1045 steel substrates were ground with sandpaper sequentially from 80# to 2000# grit, followed by thorough cleaning before use. Film deposition was carried out under a temperature range of 200~350 °C. High-purity nitrogen (N_2_, 99.999%) was used as both carrier and purge gas. The single-cycle deposition sequence consisted of: TBTDET pulse (0.05~0.5 s) → N_2_ purge (20 s) → NH_3_ pulse (1~10 s) → N_2_ purge (20 s). Importantly, for each condition, Si and AISI 1045 substrates were placed side-by-side in the ALD chamber and deposited simultaneously.

### 2.2. Preparation of ALD-TaOx Films

TBTDET and deionized water (H_2_O, 18.5 MΩ·cm) were employed as the tantalum (Ta) and oxygen (O) precursors, respectively. The substrates, carrier gas, and purge gas were identical to those used in Section 2.1. Deposition was carried out over a temperature range of 150~400 °C. The single-cycle process steps were as follows: TBTDET pulse (0.1~1 s) → N_2_ purge (20 s) → H_2_O pulse (0.01~0.03 s) → N_2_ purge (20 s).

### 2.3. Preparation of ALD-TaO_x_N_γ_ and TaN/TaO_x_ Multilayered Films

**Figure 1:**
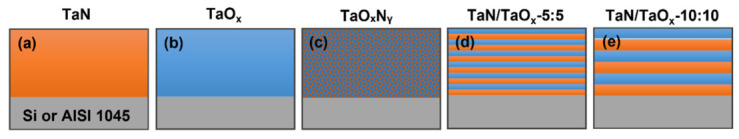
Schematic diagrams of different Ta-based films grown by ALD: (**a**) TaN, (**b**) TaO_x_, (**c**) TaO_x_N_γ_, (**d**) TaN/TaO_x_-5:5, and (**e**) TaN/TaO_x_-10:10.

Based on the optimized parameters established in Section 2.1 and Section 2.2, the growth processes for the ALD-grown homogeneous TaO_x_N_γ_ film and the multilayered TaN/TaO_x_ film were defined. The deposition temperature was set to the common processing window for both binary films, i.e., 300 °C. The optimized single-cycle process parameters were as follows: TaN sub-cycle: TBTDET pulse (0.4 s) → N_2_ purge (20 s) → NH_3_ pulse (4 s) → N_2_ purge (20 s); TaO_x_ sub-cycle: TBTDET pulse (0.3 s) → N_2_ purge (20 s) → H_2_O pulse (0.02 s) → N_2_ purge (20 s). The substrates, carrier gases, and purge gases used were identical to those described in Section 2.1. By integrating the above-optimized cycles and tuning either the sub-cycle ratio or the individual sub-layer thicknesses, homogeneous TaO_x_N_γ_ films and multilayered TaN/TaO_x_ films were grown, respectively. Specifically, for the homogeneous TaO_x_N_γ_ film, the TaN-to-TaO_x_ sub-cycle ratio (*m*:*n*) was set to 1:1; for the multilayered TaN/TaO_x_ films, the thickness ratio of TaN sub-layers to TaO_x_ sub-layers (*T*_1_:*T*_2_) was set to 5 nm:5 nm and 10 nm:10 nm. The total thickness of all the films used for electrochemical detection was controlled consistently (60 nm ± 3 nm). The schematic diagrams of prepared samples are shown in Figure 1. Noting that the TaO_x_N_γ_ film is referred to as “homogeneous”, indicating both chemically uniform composition throughout the film thickness and the absence of any periodic layered structure, confirmed by the cross-sectional TEM and EDS line scanning below.

### 2.4. Microstructure Inspection

The thickness was measured using an ellipsometry spectrometer (Alpha-SE, J. A. Woollam, Lincoln, NE, USA) to determine the growth rate. The surface morphology was examined by means of a metallographic microscope (MM, Leica DM2700 M, Wetzlar, Germany) and a scanning electron microscope (SEM, FEI Nova 430, FEI Company, Hillsboro, OR, USA, operated at an accelerating voltage of 15 kV). The phase composition and crystallinity were investigated by grazing-incidence X-ray diffraction (GIXRD, Bruker D8 Advance, Bruker AXS GmbH, Karlsruhe, Germany, Cu Kα radiation). The surface roughness was analyzed using an atomic force microscope (AFM, Bruker Dimension Icon, Santa Barbara, CA, USA). The chemical bonding states and elemental composition were evaluated using X-ray photoelectron spectroscopy (XPS, Escalab 250 Xi, Thermo Fisher Scientific, East Grinstead, UK). Prior to spectral fitting, the XPS data were calibrated based on the C 1s peak at 284.8 eV as a reference; microstructure was thoroughly characterized using a transmission electron microscope (TEM, Talos F200X, Thermo Fisher Scientific, Waltham, MA, USA).

### 2.5. Electrochemical Performance Testing

Electrochemical tests were performed at room temperature using an electrochemical workstation (CHI 760E, Shanghai Chenhua Instrument Co., Ltd., Shanghai, China). The measurements were carried out in a standard three-electrode cell configuration: the working electrode was either AISI 1045 steel or ALD film-protected sample with an exposed area of 1 cm^2^, the reference electrode was a saturated silver/silver chloride electrode (RE305 type), and the counter electrode was a platinum sheet. The electrolyte solution was 3.5 wt.% NaCl, adjusted to a pH of 7.0. In the potentiodynamic polarization experiments, the potential was scanned from −1.0 V to +1.0 V relative to the open-circuit potential at a scan rate of 1 mV/s. For electrochemical impedance spectroscopy (EIS) measurements, a sinusoidal Alternating Current (AC) perturbation of 10 mV amplitude was applied over a frequency range from 100 kHz to 0.1 Hz. The EIS curves were fitted to appropriate equivalent circuits using ZSimpWin software 3.60. All electrochemical measurements were performed on three independently prepared parallel samples for each condition.

### 2.6. Salt Spray Corrosion Test

According to the ASTM B117 standard [21], a neutral salt spray test was performed in a Yashil Salt Spray Constant Temperature and Humidity Chamber (model FGDJ-250, Beijing Yashil Testing Equipment Co., Ltd. Beijing, China). Prior to testing, an organic silicone sealant was applied around the edges of all samples to prevent environmental interference with the exposed surfaces. Experiment conditions were maintained as follows: temperature at (35 ± 2) °C, spraying duration of 2 h, 5 wt.% NaCl solution (pH = 7.0), and ambient humidity kept below or equal to 85% RH. During the test intervals (0 h, 10 min, 30 min, 1 h, 1.5 h, and 2 h), the spraying was temporarily halted to allow rapid observation and documentation of surface changes on the samples. After completion of the test, the samples were rinsed with de-ionized water, dried, and subsequently subjected to surface morphology analysis and characterization of corrosion products.

## 3. Results and Discussion

### 3.1. Growth Characteristics and Structural Component Characterization of ALD-TaN Films

**Figure 2:**
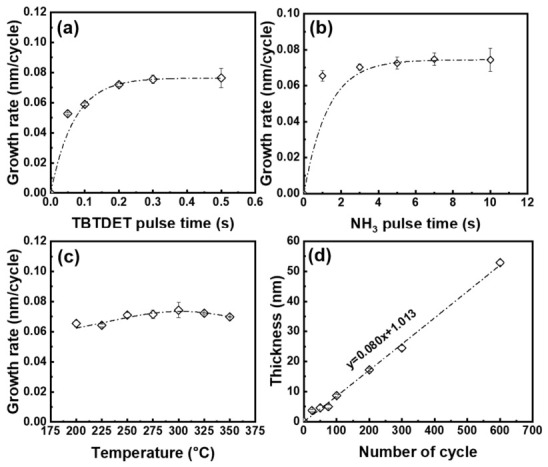
ALD-TaN film growth rate versus (**a**) TBTDET pulse time, (**b**) NH_3_ pulse time, (**c**) deposition temperature, and (**d**) film thickness as a function of deposition cycles.

Figure 2a illustrates the dependence of the TaN film growth rate on the pulse duration of the metal precursor TBTDET, under deposition conditions of 275 °C and a fixed NH_3_ pulse time of 4 s (i.e., the saturation curve). As shown in the figure, the film growth rate increased progressively with increasing TBTDET pulse duration. When the pulse time reached 0.4 s, the growth rate approached saturation, stabilizing at approximately 0.075 nm/cycle. Further extension of the TBTDET pulse duration resulted in no significant increase in the growth rate, clearly indicating that the chemisorption of TBTDET onto reaction sites reached completion within 0.4 s. Correspondingly, Figure 2b presents the saturation curve for the co-reactant NH_3_, obtained at the same deposition temperature of 275 °C with a fixed TBTDET pulse time of 0.4 s. The results demonstrated that the film growth rate attained a saturated plateau when the NH_3_ pulse time reached 4 s, with the growth rate stabilizing at approximately 0.075 nm/cycle. This saturation behavior further confirmed the self-limiting nature of NH_3_ adsorption and surface reaction on the deposited film. Based on the saturated pulse durations of the precursor (TBTDET: 0.4 s) and the co-reactant (NH_3_: 4 s), the influence of deposition temperature on growth behavior of TaN films was systematically investigated. As can be seen in Figure 2c, the growth rate remained nearly constant at approximately 0.07 nm/cycle over a broad temperature range from 200 °C to 300 °C. No significant increase in growth rate was observed with rising temperature, nor was there a sharp decline at lower temperatures across the entire studied range. This flat and wide temperature window revealed an excellent ALD process and high thermal stability for ALD-TaN film deposition. Furthermore, to evaluate the linear controllability of film growth and nucleation characteristics, deposition experiments with varying cycle numbers (ranging from 25 to 600 cycles) were performed under optimized conditions at 275 °C, as shown in Figure 2d. The experimental data were subjected to linear regression analysis, yielding a slope of approximately 0.08. This fitted value was in close agreement with both the precursor saturation-derived growth rate and the steady-state growth rate of 0.075 nm/cycle measured within the ALD window. More importantly, no discernible nucleation delay was detected in the low-cycle regime, further confirming that the TaN films grow in a highly linear, layer-by-layer manner under these ALD process conditions [22].

**Figure 3:**
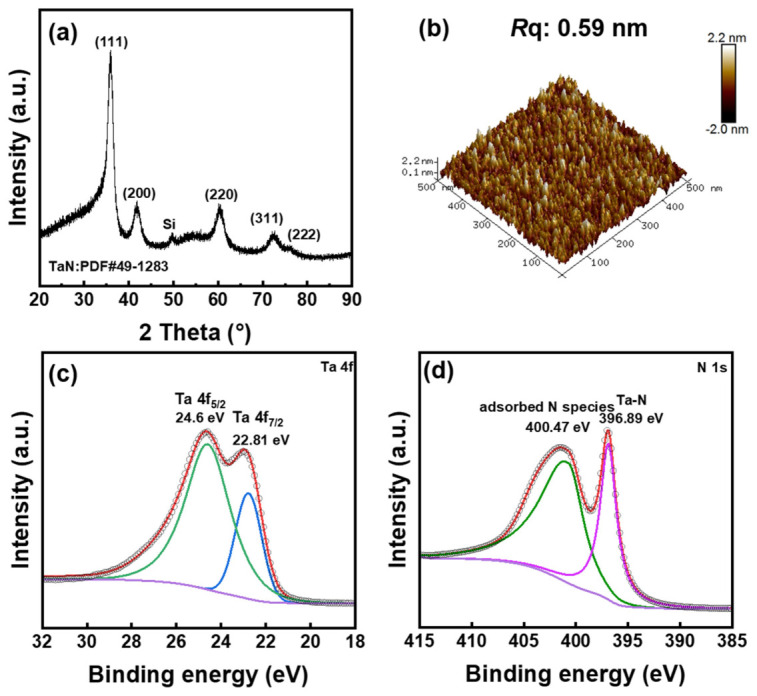
(**a**) GIXRD patterns, (**b**) AFM surface morphology and (**c**,**d**) XPS patterns of ALD-TaN thin films.

Figure 3 presents the GIXRD pattern, AFM morphology, and XPS analysis results of the TaN film deposited at 275 °C. From the XRD analysis (Figure 3a), distinct diffraction peaks were observed at 35.8°, 41.6°, 60.3°, 72.1°, and 75.9°, which could be indexed to the (111), (200), (220), (311), and (222) crystal planes of the face-centered cubic (fcc) structure of TaN, as referenced in the standard PDF card (PDF #49-1283). However, the diffraction peaks exhibit significant broadening. The full width at half maximum (FWHM) of the TaN (200) peak was calculated using the Scherrer Formula (1), and the average grain size was approximately 8–12 nm, indicating that this film has a nanocrystalline structure.

(1) D=kλ/(βcosθ)

Among them, *D* represents the grain size, *k* is the Scherrer constant, *λ* is the X-ray wavelength, *β* is the full width at half maximum (FWHM) of the diffraction peak, and *θ* is the diffraction angle [23]. In addition to a weak signal originating from the Si substrate (around 69°), no diffraction peaks corresponding to any secondary or impurity phases were detected, indicating the absence of detectable secondary or impurity phases, i.e., high phase purity. From the AFM analysis (Figure 3b), it was evident that the TaN films exhibit excellent surface flatness and uniformity, with root-mean-square roughness (*R*q) values consistently below 0.6 nm. Both AFM and GIXRD analyses indicated that ALD-grown TaN films using the TBTDET/NH_3_ were dense and uniform fcc structure over a broad temperature range. Figure 3c,d present the XPS analysis of the TaN films. In the Ta 4f core-level spectrum, a characteristic spin–orbit doublet was observed, with binding energies at 22.8 eV (Ta 4f_7/2_) and 24.7 eV (Ta 4f_5/2_), yielding a splitting energy of 1.9 eV. The intensity ratio of the doublet closely matched the theoretical value of 4:3. These binding energy values were consistent with those reported for cubic-phase TaN, confirming that tantalum predominantly existed in the nitrided state [24]. The N 1s spectrum can be deconvoluted into two components: a dominant peak at 396.9 eV, attributed to lattice nitrogen in Ta–N bonds, and a weaker shoulder at 400.5 eV, corresponding to surface-adsorbed nitrogen-containing species (e.g., amines, nitriles). The latter might arise from residual traces of incompletely decomposed precursor ligands or environmental contamination [25].

**Figure 4:**
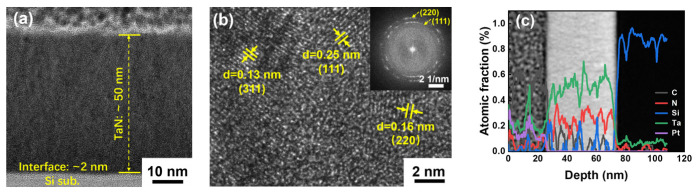
TEM analysis of the cross-section of ALD-TaN thin films: (**a**) Overall microscopic morphology, (**b**) high-resolution image, (**c**) Cross-sectional EDS analysis.

Figure 4 presents the TEM analysis of the TaN film deposited at 275 °C. As shown in Figure 4a, the film exhibited uniform thickness, approximately 50 nm, and displayed a high-density nanocrystalline structure. A ~2 nm-thick amorphous transition layer was observed at the interface between the substrate and the film, with minimal interfacial fluctuation, indicating atomic-level flatness. The formation of this transition layer might be attributed to two possible factors: (1) the reaction between the native oxide layer on the Si substrate (SiO_x_) and the initial TaN interface products (e.g., tantalum silicate); and (2) the amorphous growth behavior during the initial ALD nucleation stage [26]. High-resolution TEM (HRTEM) images in Figure 4b clearly reveal the (111), (200), and (311) lattice fringes, with measured interplanar spacings of d(111) = 0.245 nm, d(200) = 0.212 nm, and d(311) = 0.151 nm, respectively, which are highly consistent with the XRD results discussed earlier. The Fast Fourier Transform (FFT) pattern (inset) exhibited well-defined polycrystalline diffraction rings, corresponding sequentially to the fcc-TaN (111) and (220) reflections. The low dispersion of diffraction spots further confirmed that the ALD-grown TaN film possessed a highly ordered short-range nanocrystalline structure. High-angle annular dark-field scanning transmission electron microscopy (HAADF-STEM) combined with EDS (Figure 4c) analysis revealed that the Ta and N elements were highly uniformly distributed within the film matrix, whereas a sharp elemental concentration gradient was observed at the interface, where the Ta/N signal decreased to background levels within a 2 nm transition layer. The carbon impurity content was found to be very low (<3 at%), which was fully corroborated by the XPS analysis results.

### 3.2. Growth Characteristics and Structural Component Characterization of ALD-TaOx Thin Films

**Figure 5:**
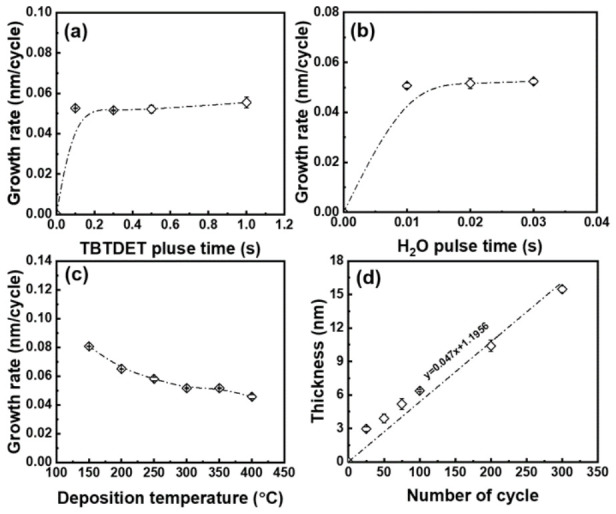
ALD-TaO_x_ film growth rate versus (**a**) TBTDET pulse time, (**b**) H_2_O pulse time, (**c**) deposition temperature, and (**d**) film thickness as a function of deposition cycles.

Figure 5 systematically exhibits the growth characteristics of TaO_x_ films using TBTDET and H_2_O. The saturation behavior of the TBTDET precursor is illustrated in Figure 5a. As shown, the growth rate of the TaO_x_ film presented a typical adsorption saturation trend with increasing TBTDET pulse duration. The growth rate reached a plateau at approximately 0.05 nm/cycle when the pulse time was ≥0.1 s, indicating complete surface saturation of the Ta precursor on the substrate’s active sites. This growth rate was notably lower than that observed for TaN under identical conditions (0.07 nm/cycle), suggesting a higher energy barrier associated with ligand exchange during the formation of Ta-based oxides compared to nitrides [27]. The saturation curve of the H_2_O co-reactant in Figure 5b demonstrated that growth saturation was achieved at a H_2_O pulse time of ≥0.01 s, yielding a consistent growth rate of ~0.05 nm/cycle. The rapid saturation indicates high reactivity of water molecules in the formation of Ta-based oxides. This could be attributed to the relatively low energy barrier for proton transfer reactions on the Ta–OH* surface, which was significantly lower than that required for N–H bond dissociation in NH_3_, thereby facilitating faster surface saturation [28]. Under optimized saturation pulse time conditions, the ALD window was investigated across a range of deposition temperatures (Figure 5c). Within the temperature range of 150~400 °C, the growth behavior of TaO_x_ films exhibited a three-stage characteristic: (1) The low-temperature regime (150~250 °C): the growth rate decreased from 0.07 nm/cycle to 0.05 nm/cycle, which can be attributed to the weakening of precursor physisorption as temperature increased. (2) The steady-state regime (250~350 °C): the growth rate remained approximately constant at 0.05 nm/cycle, indicating that the surface reaction was governed by self-limiting chemisorption. (3) The high-temperature regime (>350 °C): the growth rate further declined (reaching ~0.03 nm/cycle at 400 °C), likely due to competitive desorption of H_2_O molecules on the activated surface [29]. The 100-wide range window (250~350 °C) demonstrates that the TaO_x_ films grown via this process exhibit an ALD window comparable to that of the previously described TaN films. Similarly, a linear growth experiment was performed at a deposition temperature of 275 °C, as shown in Figure 5d. The film thickness showed a highly linear correlation with the number of deposition cycles, yielding a fitted slope of approximately 0.047 nm/cycle, which was in excellent agreement with the saturation growth rate derived from the preceding analysis. Furthermore, during the initial growth stage (the first 25 cycles), a slight super-linear growth trend was observed, suggesting high reactivity of surface -OH groups at the early deposition stage.

**Figure 6:**
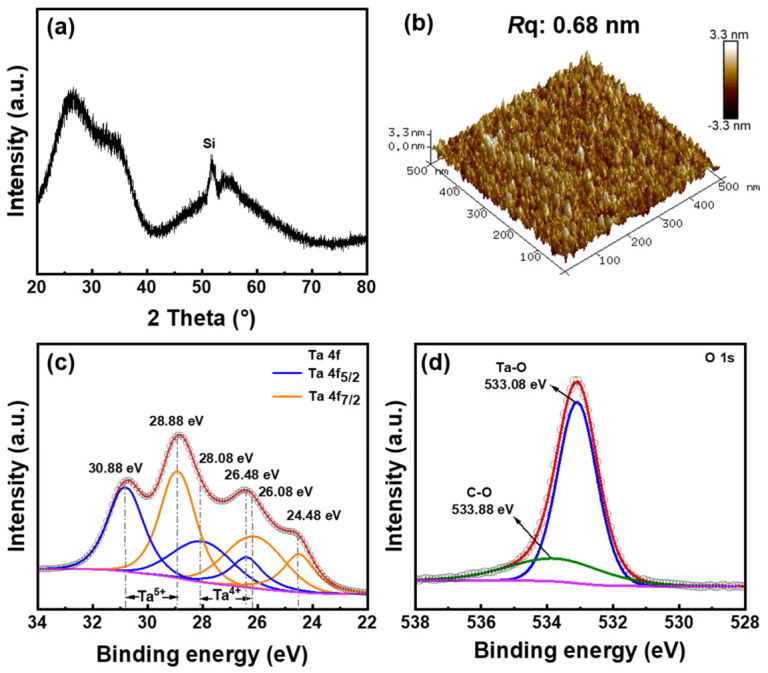
(**a**) GIXRD patterns, (**b**) AFM surface morphology and (**c**,**d**) XPS patterns of ALD-TaO_x_ thin films.

Figure 6 comprehensively illustrates the microstructure and chemical characteristics of the TaO_x_ film fabricated at 275 °C. The XRD pattern (Figure 6a) exhibited no sharp diffraction peaks, indicating that the film was amorphous in nature. AFM morphological analysis (Figure 6b) further confirmed excellent surface continuity, with a root mean square roughness (*R*q) of approximately 0.68 nm. In the XPS analysis, the Ta 4f high-resolution spectrum (Figure 6c) revealed three distinct chemical states: the dominant Ta^5+^ state (4f_7_/_2_: 26.8 eV) corresponds to the stoichiometric Ta_2_O_5_ phase; the Ta^4+^ state (4f_7_/_2_: 25.2 eV) originated from local structural defects within the amorphous matrix, such as oxygen vacancies or coordination-unsaturated tantalum sites, rather than from classical grain boundaries which were absent in amorphous films. A minor component at approximately 22.1 eV was observed, which was tentatively attributed to oxygen-vacancy-related defects or partially reduced tantalum species within the amorphous matrix [30,31]. Meanwhile, the O 1s spectrum (Figure 6d) could be deconvoluted into three contributions: lattice oxygen (Ta–O, 530.5 eV), weakly bonded oxygen in amorphous regions (Ta–O–H, 532.0 eV), and surface-adsorbed C–O species (533.7 eV, accounting for ~10%) [32].

**Figure 7:**
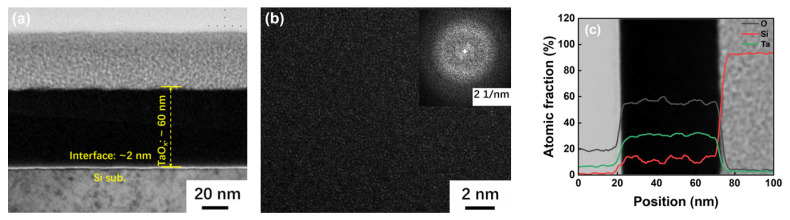
TEM analysis of the cross-sectional ALD-TaO_x_ thin films: (**a**) Overall microscopic morphology, (**b**) high-resolution image, (**c**) cross-sectional energy spectrum analysis.

TEM analysis further confirmed (Figure 7) that the TaO_x_ film deposited at 275 °C exhibited typical features of an amorphous structure. No periodic lattice fringes were observed in the HRTEM image, and the FFT pattern displayed a diffuse halo (inset in Figure 7b), collectively indicating a long-range disordered atomic arrangement. EDS mapping (Figure 7c) further confirmed that Ta and O were uniformly distributed throughout the film thickness, consistent with the self-limiting, layer-by-layer growth mechanism of ALD. At the interface region, the Si signal increased sharply, becoming dominant toward the substrate, while the Ta/O signal ratio decreased to background levels, presenting a very thin and well-defined interface.

### 3.3. Structural and Compositional Analysis of ALD-TaOxN_γ_ and TaN/TaOx Multilayered Films

**Figure 8:**
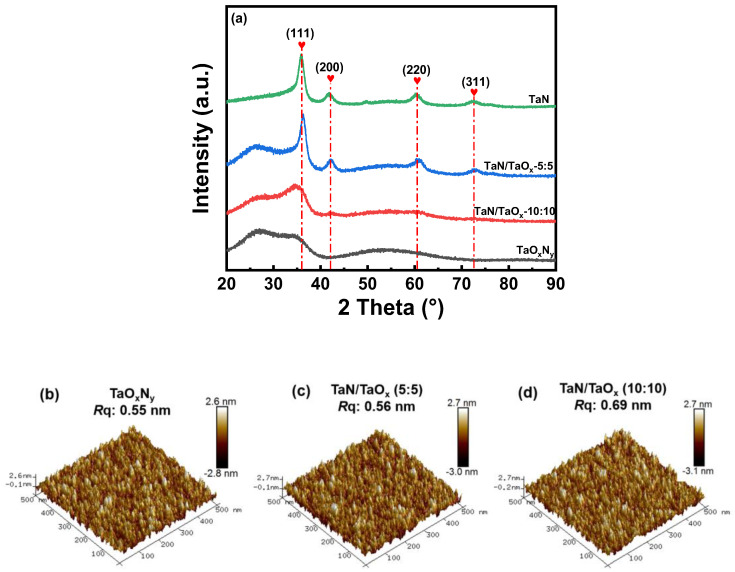
(**a**) GIXRD patterns and AFM surface morphology of ALD-Ta based films: (**b**) TaO_x_N_γ_, (**c**) TaN/TaO_x_-5:5 and (**d**) TaN/TaO_x_-10:10).

Based on the previously optimized ALD-TaN (ALD window: 200~300 °C) and ALD-TaO_x_ (ALD window: 250~350 °C) film processing techniques, homogeneous TaO_x_N_γ_ films and TaN/TaO_x_ multilayered films were successfully fabricated by precisely controlling the sub-cycle ratio of TaN and TaO_x_. Figure 8 systematically illustrates the evolution of crystal structure and surface morphology in the composite films, with the observed structural changes profoundly reflecting the significant influence of compositional variations and interfacial effects on the film growth mechanism. As shown in Figure 8a, the incorporation of TaO_x_ components exerted a decisive impact on the crystallinity of the films. In the multilayered film (TaN/TaO_x_-10:10), although the primary XRD diffraction peaks were still attributable to the TaN phase, their positions exhibited a systematic shift toward higher angles (approximately 0.18°). This shift was considered to be probably from a combination of compressive lattice distortion induced by the adjacent amorphous TaO_x_ layers and oxygen incorporation from the TaO_x_ sublayers into the TaN lattice, which partially substitutes nitrogen with the smaller oxygen atoms [33]. When the sub-cycle ratio was reduced to 5:5 (TaN/TaO_x_-5:5), the amorphous TaO_x_ significantly impeded the continuous growth of TaN grains, resulting in an overall amorphous film. The homogeneous TaO_x_N_γ_ film synthesized at a 1:1 cycle ratio exhibited a completely amorphous structure, revealing that during the atomic-scale alternating deposition process, oxygen and nitrogen atoms achieved a high degree of miscibility, effectively suppressing the nucleation and growth of any crystalline phase and thereby forming a unique amorphous solid solution [34]. Further AFM characterization (Figure 8b–d) revealed that the homogeneous TaO_x_N_γ_ film and the two multilayered films exhibited exceptionally low surface roughness, with root-mean-square (*R*q) values of 0.55 nm for TaO_x_N_γ_, 0.56 nm for TaN/TaO_x_-5:5, and 0.69 nm for TaN/TaO_x_-10:10, attributed to the precise atomic-level thickness control and superior interface flatness of the ALD process. The atomically smooth surface of these films is critical for their effectiveness as protective layers, particularly in suppressing the penetration of corrosive species at defect sites.

**Figure 9:**
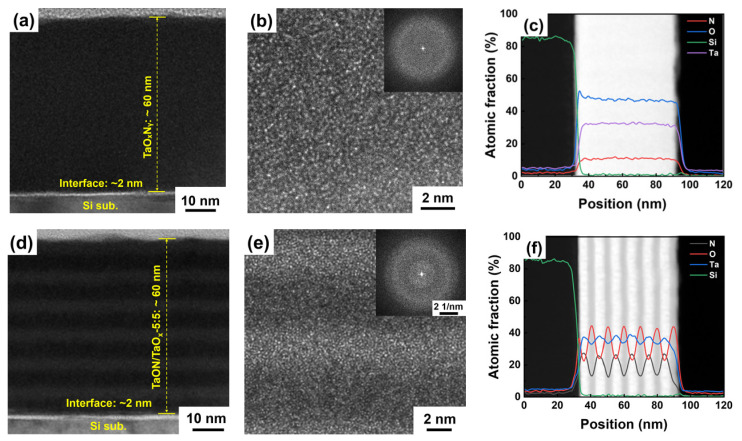
TEM bright-field images, high-resolution image, FFT and energy spectrum depth analysis of (**a**–**c**) homogeneous TaO_x_N_γ_ films and (**d**–**f**) TaN/TaO_x_-5:5 nanoscale multilayers.

Furthermore, HRTEM and elemental depth profiling were employed to elucidate the structural characteristics of the homogeneous TaO_x_N_γ_ and the TaN/TaO_x_-5:5 films (Figure 9). In the homogeneous TaO_x_N_γ_ system, neither lattice fringes nor diffraction spots were observed in the TEM bright-field image or the FFT, respectively, confirming its fully amorphous nature. This observation was fully corroborated by the prior XRD results. It fundamentally arises from the high miscibility between TaN and TaO_x_; the rapid interdiffusion of O and N atoms at the sub-cycle interfaces suppresses long-range lattice ordering. Furthermore, EDS line scanning revealed that the three elements (Ta, O, and N) were uniformly distributed throughout the entire film thickness, with no detectable compositional gradients or layered heterogeneity. For the TaN/TaO_x_-5:5 sample, cross-sectional TEM clearly exhibited a layered structure with alternating bright and dark contrast (period: 5 nm), thereby providing direct evidence of ALD’s precise capability in constructing nanoscale heterogeneous interfaces. No lattice fringes were observed in the high-resolution images and the FFT patterns, indicating that the multilayer film retained an amorphous composite structure. In this architecture, the amorphous TaO_x_ layer effectively suppressed the grain growth of TaN, promoting independent nucleation within each sublayer. Moreover, EDS line scanning demonstrated that the signals of Ta, O, and N exhibited periodic stripes along the depth direction, with the spatial positions of the O and N peaks precisely corresponding to the TaO_x_ and TaN layers, respectively.

**Figure 10:**
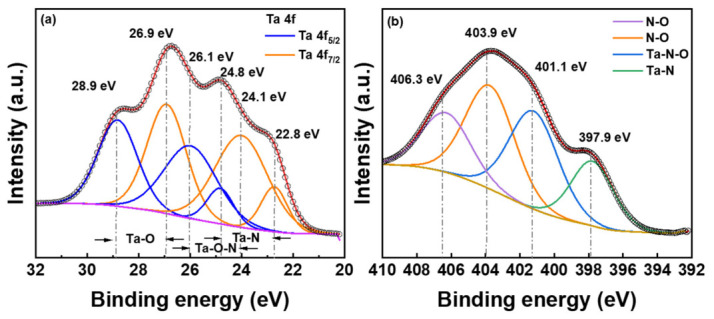
XPS patterns of ALD-TaO_x_N_γ_ thin films: (**a**) Ta 4f, (**b**) N 1s.

To understand the chemical bonding environment in the homogeneous TaO_x_N_γ_ film, XPS analysis was performed, and the results are presented in Figure 10a,b. The Ta 4f spectrum of the TaO_x_N_γ_ film (Figure 10a) was deconvoluted into three doublets. The lowest binding energy doublet (Ta 4f_7_/_2_ at 22.8 eV) corresponds to Ta–N bonds, similar to those in TaN. The highest binding energy doublet (26.9 eV) corresponds to Ta–O bonds, as in Ta_2_O_5_. Crucially, an intermediate doublet at 24.1 eV (Ta 4f_7_/_2_) is assigned to Ta–O–N oxynitride species, where tantalum is simultaneously bonded to both oxygen and nitrogen. This indicates the formation of a true ternary amorphous phase rather than a simple mixture of TaN and TaO_x_ domains. The N 1s spectrum (Figure 10b) further supports this, with peaks at 397.9 eV (Ta–N) and 401.1 eV (Ta–O–N). The presence of mixed Ta–O–N bonding introduces both ionic (Ta–O) and covalent (Ta–N) character, which increases structural disorder, eliminates grain boundaries, and enhances chemical inertness. This unique bonding environment is believed to be the origin of the superior corrosion resistance of the TaO_x_N_γ_ film [35]. Quantitative XPS analysis of the TaO_x_N_γ_ film yields O/Ta and N/Ta atomic ratios of approximately 0.81 and 0.38, respectively, confirming the oxynitride stoichiometry (Table S1). The above findings provided direct evidence for the successful fabrication of an amorphous, uniform ternary Ta-based film, which was considered to underpin the superior corrosion resistance by eliminating grain boundaries and adopting a highly disordered atomic structure.

### 3.4. Study on the Electrochemical Corrosion Behavior of Ta-Based Films

#### 3.4.1. Polarization Analysis by Potentiodynamic Method

**Figure 11:**
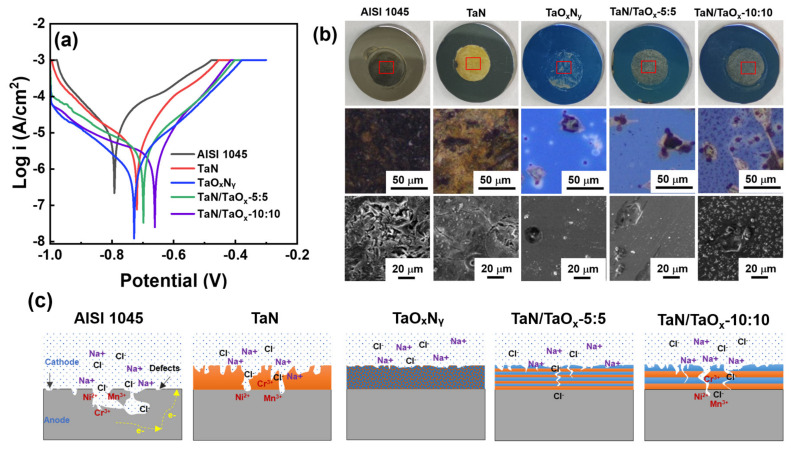
(**a**) Polarization curves of AISI 1045 matrix and ALD-grown Ta-based film-coated samples (TaN, TaO_x_N_γ_, TaN/TaOx-5:5, TaN/TaOx-10:10) in 3.5 wt.% NaCl solution. (**b**) Corrosion morphology after electrochemical testing. (**c**) Schematic diagram illustrating the proposed corrosion mechanisms based on electrochemical and salt spray results. It is noted that direct visualization of Cl^−^ penetration pathways (e.g., by SIMS) was not performed, and the proposed mechanisms remain speculative.

Figure 11 presents the electrochemical polarization behavior and corrosion morphology of AISI 1045 steel substrate and four ALD-grown Ta-based film-coated samples (TaN, TaO_x_N_γ_, TaN/TaO_x_-5:5, TaN/TaO_x_-10:10) in a 3.5 wt.% NaCl solution. The corrosion potential (*E*_corr_) and corrosion current density (*I*_corr_), listed in Table 1, were determined using the Tafel extrapolation method according to the polarization curves shown in Figure 11a. As presented, none of the polarization curves exhibited a distinct passivation plateau. Although tantalum oxynitrides are known to be inherently passive, the absence of such a plateau can be attributed to three interrelated factors. First, the ~60 nm ALD films were approximately two orders of magnitude thinner than conventional passivation layers, resulting in a dominant leakage current that obscured any passivation signature. Second, the high chloride ion concentration (3.5 wt.%) promoted localized pitting corrosion, wherein local anodic currents overwhelmed and masked any measurable passivation response. Third, corrosion protection was primarily afforded not by the formation of a thick, electrochemically passive oxide film, but rather by a dense, physically robust barrier that effectively suppressed chloride ion diffusion into the underlying substrate. Consequently, the observed electrochemical behavior did not compromise the material’s exceptional corrosion resistance, but instead reflected its distinctive, barrier-dominated protection mechanism. For the bare AISI 1045 steel substrate, the corrosion potential was −0.82 V, and the corrosion current density reached as high as 1.61 × 10^−5^ A·cm^−2^. After surface protection with ALD films, the corrosion potentials of all coated samples shifted positively by 0.07~0.11 V, and the corrosion current densities decreased by approximately one order of magnitude, down to the range of 10^−6^ A·cm^−2^. Among the coated samples, the homogeneous TaO_x_N_γ_ film exhibited the most positive corrosion potential (approximately −0.75 V) and the lowest corrosion current density (approximately 1.20 × 10^−6^ A·cm^−2^). A quantitative comparison with representative ALD coatings reported in the literature (summarized in Table S2 [11,16,36,37,38]) revealed that our TaO_x_N_γ_ film exhibited superior corrosion resistance, with a markedly lower *E*_corr_ and *I*_corr_ than most previously reported ALD-grown protective layers. This superior performance was attributed to the dense, amorphous microstructure of the film, which effectively hindered the penetration of Cl^−^ ions. For multilayered films, the corrosion current densities of TaN/TaO_x_-5:5 and TaN/TaO_x_-10:10 were comparable; however, the corrosion potential of TaN/TaO_x_-5:5 was relatively higher, at approximately −0.71 V. The corrosion morphology (Figure 11b) further corroborated these findings: the AISI 1045 steel substrate exhibited pronounced corrosion features, with a high density of corrosion pits formed on its surface. After being protected by the TaN film, microcracks and localized pitting corrosion were observed, indicating inadequate protective performance. Although no obvious corrosion-induced cracks formed in either the multilayer-protected or the homogeneous TaO_x_N_γ_-protected samples, they exhibited distinct corrosion damage morphologies. The surface of TaN/TaO_x_-5:5 contained larger corrosion pits, whereas TaN/TaO_x_-10:10 showed not only corrosion pits but also structural degradation of the protective layer. In contrast, the homogeneous TaO_x_N_γ_ sample exhibited only minimal traces of pitting, with no evident structural alteration to the film, further confirming its superior corrosion resistance. Based on the corrosion behavior revealed by the polarization curves presented above, Figure 11c schematically illustrates the surface degradation process of the different samples under chloride (Cl^−^) attack. The proposed Cl^−^ diffusion pathways and the interfacial blocking effect depicted in the figure are hypothetical mechanisms formulated on the basis of electrochemical measurements and salt spray test results. The AISI 1045 substrate exhibited extensive surface damage, with numerous penetrating corrosion pits extending deep into the material. Although the TaN coating provided a certain degree of protection and mitigated substrate corrosion, it suffered significant degradation itself, as evidenced by the presence of microcracks and pitting corrosion on its surface. In contrast, the homogeneous TaO_x_N_γ_ film showed only minor surface erosion, while the underlying steel substrate remained unaffected, indicating superior barrier properties. For multilayer structures, the TaN/TaO_x_ interface effectively impeded Cl^−^ ion migration; however, the protective performance was highly dependent on the sublayer thickness. When the total coating thickness was constant, reducing the individual sublayer thickness could increase the interface density, thereby significantly enhancing corrosion resistance through improved inhibition of ion transport.

#### 3.4.2. EIS Analysis

**Figure 12:**
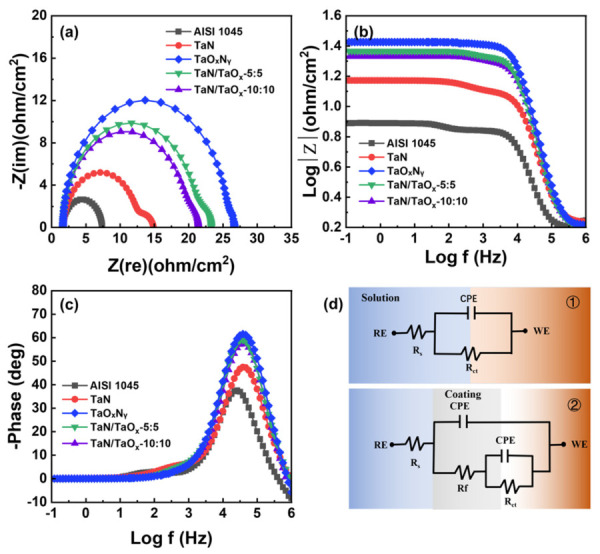
EIS analysis results of AISI 1045 substrate and four ALD-grown Ta-based film-coated samples (TaN, TaO_x_N_γ_, TaN/TaO_x_-5:5, TaN/TaO_x_-10:10) in 3.5 wt.% NaCl solution: (**a**) Nyquist plot, (**b**) Bode amplitude plot, (**c**) Bode phase plot and (**d**) equivalent circuit design.

Figure 12 presents the EIS results for AISI 1045 steel substrate and samples coated with TaN, TaO_x_N_γ_, TaN/TaO_x_-5:5, and TaN/TaO_x_-10:10 in a 3.5 wt.% NaCl solution at pH 7. The results included Nyquist plots, Bode magnitude plots, Bode phase angle plots, and corresponding equivalent circuit models. The detailed fitting parameters are summarized in Table 2 and Table 3.

As can be observed from the Nyquist plot (Figure 12a), the AISI 1045 steel substrate exhibited a single capacitive loop, represented by a 45° inclined line in the low-frequency region, which was indicative of a diffusion-controlled dissolution process governed by Cl^−^ diffusion. In contrast, all ALD-deposited protective coatings displayed a double capacitive loop feature, confirming the presence of a dual-layer protective structure at the film–metal interface. Compared to the bare AISI 1045 steel substrate, all coated samples demonstrated significantly larger diameters of the capacitive arcs, reflecting an enhanced charge transfer resistance (*R*_ct_). To quantitatively interpret the EIS responses, two equivalent circuit models were employed, as shown in Figure 12d. For the bare AISI 1045 steel substrate (Model ①), a circuit consisting of *R*_s_, *CPE*, *R*_ct_, and an inductance *L* was adopted. Here, *R*_s_ represents the solution resistance, *Q*_dl_ is the constant phase element (CPE) accounting for the non-ideal capacitive behavior of the electrical double layer, and *R*_ct_ denotes the charge transfer resistance. The inductance L was introduced to fit the low-frequency inductive loop, which is typically associated with metastable pitting corrosion induced by Cl^−^ ions [39]. For all ALD-coated samples (Model ②), an additional high-frequency time constant was observed, corresponding to the response of the protective coating. Accordingly, Model ② was derived by adding a parallel *R*_f_//*Q*_f_ branch to the high-frequency region of Model ①. In this branch, *R*_f_ represents the coating resistance (also referred to as pore resistance), reflecting the ionic permeability and barrier integrity of the ALD layer, while *Q*_f_ is a *CPE* describing the non-ideal capacitive behavior of the coating itself [40]. The trend in arc diameter followed the sequence: *R*_ct_ (TaO_x_N_γ_) > *R*_ct_ (TaN/TaOx-5:5) > *R*_ct_ (TaN/TaO_x_-10:10) > *R*_ct_ (TaN) > *R*_ct_ (AISI 1045), which was in strict agreement with the *R*_ct_ values derived from equivalent circuit fitting (Table 2). Notably, the TaO_x_N_γ_-coated sample exhibited the largest capacitive arc diameter, with an *R*_ct_ value of 24.75 Ω·cm^2^, markedly higher than that of the uncoated substrate, demonstrating that the homogeneous and dense TaO_x_N_γ_ passivation layer provided the most effective barrier against Cl^−^ ion penetration and thus offered superior corrosion resistance. For the multilayered coatings, the TaN/TaO_x_-5:5 sample showed a higher *R*_ct_ (19.76 Ω·cm^2^) than that (18.17 Ω·cm^2^) of the TaN/TaO_x_-10:10 sample, suggesting that reducing sublayer thickness could contribute to improving corrosion protection within a multilayer architecture.

The Bode amplitude plot (Figure 12b) in the frequency range of 0.1 Hz to 3.16 kHz revealed that the log|Z| values of all films remained nearly constant (TaO_x_N_γ_: 1.41, TaN/TaO_x_-5:5: 1.39, TaN/TaO_x_-10:10: 1.35, TaN: 1.19), indicating the absence of coating defects that would allow electrolyte permeation and effective suppression of charge transfer reactions in all samples. Among these, the TaO_x_N_γ_ film, possessing a homogeneous amorphous structure, maximized the complexity of the Cl^−^ diffusion pathway, thereby exhibiting the best barrier protection performance (log|Z| ≈ 1.41, i.e., |Z| = 2.57 × 10^5^ Ω·cm^2^). For the multilayer nano-stack structures, the log|Z| value of TaN/TaO_x_-5:5 was approximately 1.39 (|Z| = 2.45 × 10^5^ Ω·cm^2^), slightly higher than that (log|Z| = 1.35, |Z| = 22.9 × 10^4^ Ω·cm^2^) of TaN/TaO_x_-10:10, suggesting superior barrier performance for the TaN/TaO_x_-5:5 configuration. This observation further indicated that reducing sublayer thickness in multilayer films could enhance their protective capability. For pure TaN, it exhibited relatively lower protection efficacy, likely due to its inherent electrical conductivity, yet it still demonstrated a significant improvement compared to the bare substrate. At frequencies above 3.16 kHz, the log|Z| values of all samples decreased simultaneously to approximately 0.2 (|Z| = 1.58 Ω·cm^2^), implying that the solution resistance (*R*s) in the system was consistent across measurements. This result was in excellent agreement with the *R*_s_ values derived from equivalent circuit fitting (1.54–1.73 Ω·cm^2^). Furthermore, the Bode amplitude plot indicated that the chemical homogeneity of the single-phase TaO_x_N_γ_ film was more effective in enhancing low-frequency impedance than the multilayer architectures.

Figure 12c shows the Bode phase diagrams of different samples. In the low-frequency region (log f: −1~3 Hz), the phase angle of all samples was approximately equal to 0°, indicating that the interface process was dominated by pure resistive behavior at this stage, with no local electrochemical corrosion. In the mid-high frequency region (phase peak characteristics: log f: 3~4.55 Hz), the Phase values of all samples gradually increased and reached a peak. The AISI 1045 matrix peaked at a log (f/Hz) value of 4.4, while other samples with protective films peaked at a log (f/Hz) value of 4.55. The phase peak ranking was: TaO_x_N_γ_ (62°) > TaN/TaO_x_-5:5 (59°) > TaN/TaO_x_-10:10 (58°) >> matrix (38°), which was consistent with the protective film efficacy. Among them, TaO_x_N_γ_ had the highest phase angle (approaching the ideal capacitance threshold of 65°) and the largest low-frequency |Z| value (2.57 × 10^5^ Ω·cm^2^), which approached the behavior of an ideal passivation film [41]. The phase angle of the multilayer film TaN/TaOx-5:5 was 1° higher than that of TaN/TaOx-10:10, suggesting that it had better interface bonding. The phase angle of the AISI 1045 steel matrix at 38° indicated that the pure matrix in the electrochemical process exhibited the characteristics of porous corrosion products.

### 3.5. Evaluation of the Neutral Salt Spray Corrosion Performance of Ta-Based Films

In order to further investigate the corrosion resistance of the various coatings, a salt spray test was performed on AISI 1045 steel, as well as the coated samples with TaN, TaO_x_N_γ_, TaN/TaO_x_-5:5, and TaN/TaO_x_-10:10 films. Prior to testing, an organic silicone sealant was applied around the edges of all samples to prevent environmental interference with the exposed surfaces. During the experiment, the surface conditions were periodically photographed and documented. After completion of the test, the samples were rinsed with deionized water, dried, and subsequently subjected to surface morphology analysis and characterization of corrosion products.

**Figure 13:**
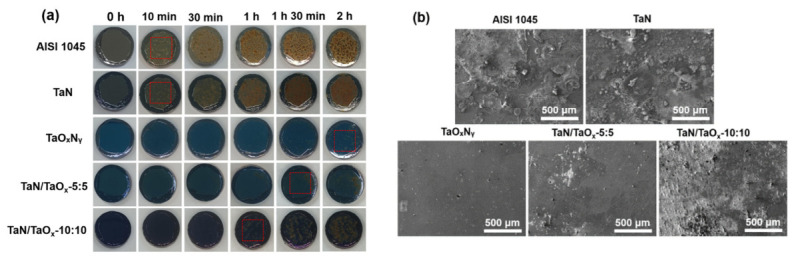
Comparison of salt spray experiments on AISI 1045 substrate and four ALD-grown Ta-based film-coated samples (TaN, TaO_x_N_γ_, TaN/TaO_x_-5:5, TaN/TaO_x_-10:10): (**a**) macroscopic morphology image, (**b**) SEM image.

Figure 13 illustrates the aging failure behavior of the AISI 1045 substrate and four protective coatings under an accelerated salt spray environment. At the initial stage of exposure (0 h), all samples exhibited a metallic lustrous surface without visible defects. After 10 min of salt spray exposure, light brown oxide spots began to appear on the surface of the AISI 1045 substrate and the TaN film, whereas the TaO_x_N_γ_ and multilayer films remained visually intact. This observation was highly consistent with the cathodic response observed in the polarization curves, as TaN possessed intrinsic electrical conductivity (conductivity > 10^3^ S/cm) [42], which probably promoted the oxygen reduction reaction and accelerated the breakdown of the passivation layer. Upon extending the exposure time to 1 h, the substrate and TaN-coated surfaces were entirely covered with reddish-brown FeOOH corrosion products. At this stage, the TaN/TaOx-10:10 sample started to exhibit a few isolated rust spots, followed by sporadic rust formation on the TaN/TaOx-5:5 sample after 1.5 h. These observations reflected progressive degradation at the interfaces of the multilayer films due to continuous penetration of the electrolyte film. The differences in aging resistance were in strict agreement with the Rct values derived from EIS, where TaN/TaOx-5:5 exhibited a higher Rf value (1.904 Ω·cm^2^) than that (1.601 Ω·cm^2^) of TaN/TaOx-10:10. After 2 h of salt spray testing, the TaOxN_γ_ film began to show a limited number of light etching spots; however, no deep red rust originating from the underlying steel substrate was observed. This indicated that, at this stage, the corrosive attack had not yet penetrated through the coating to reach the substrate. Figure 13b presents a morphological comparison of the critical point of corrosion observed in the salt spray test. For the AISI 1045 substrate and the TaN-coated sample, a substantial layer of rust products formed on the surface, revealing the underlying active dissolution behavior. The multilayer-structured film TaN/TaOx-10:10 exhibited severe damage characterized by extensive film delamination, whereas TaN/TaOx-5:5 showed only localized film degradation accompanied by a minor amount of rust formation. In contrast, the TaOxN_γ_ film displayed only slight pitting corrosion, with the majority of the coating remaining intact and effectively protective, further confirming the superior protective performance of the homogeneous TaOxN_γ_ film. It is noted that the 2 h salt spray duration used here provides a rapid comparative screening of barrier integrity under accelerated conditions. According to ASTM B117, longer exposures (24~500 h) are typically required to evaluate long-term coating durability. The present 2 h results should be interpreted as a qualitative ranking of initial corrosion resistance, not as a measure of long-term protection.

### 3.6. Analysis of Corrosion Mechanism

This study, through electrochemical testing and salt spray experiments, confirms the corrosion resistance order of the ALD-Ta-based films as TaO_x_N_γ_ > TaN/TaO_x_ multilayer > TaN. The excellent corrosion resistance of the TaO_x_N_γ_-coated samples was attributed to: (1) Amorphous structure eliminates rapid diffusion channels. The homogeneous TaO_x_N_γ_ film exhibited a fully amorphous solid solution structure (evidenced by XRD and TEM), which completely eliminated grain boundaries and pore defects, thereby maximizing the blocking of Cl^−^ diffusion (low-frequency |Z| = 2.57 × 10^7^ Ω·cm^2^). (2) Extremely low defect density enhanced barrier integrity. The dense, pinhole-free morphology achieved by ALD, combined with an ultra-smooth surface (*R*q = 0.55 nm), provided a near-perfect physical barrier that forces Cl^−^ ions to traverse a highly disordered and tortuous diffusion pathway. In contrast, polycrystalline TaN film possessed intrinsic grain boundary networks and high electrical conductivity (>10^3^ S/cm) [42], which accelerated electron transport and promote cathodic oxygen reduction, leading to early brown spot formation within 10 min under salt spray testing. (3) Multilayer architectures relied on interfacial scattering but introduced stress-related defects. For the TaN/TaO_x_-5:5 structure, reducing sublayer thickness to 5 nm doubled the interfacial density, forcing Cl^−^ ions to repeatedly cross heterointerface barriers, as reflected by the higher *R*_ct_ (19.76 Ω·cm^2^) compared to the 10:10 structure (18.17 Ω·cm^2^). However, thicker sublayers (e.g., 10:10) introduced cumulative residual stress, lattice mismatch, and possible pinhole defects, which could lead to local peeling or microcracks that became preferential corrosion initiation sites. In summary, while all coatings degraded via localized Cl^−^ accumulation, the amorphous and chemically homogeneous TaO_x_N_γ_ film presented the highest diffusion barrier, whereas multilayer films with excessively thick sublayers diminished interface coupling efficiency and rendered grain boundaries as fast diffusion paths.

## 4. Conclusions

In this study, TaN, homogeneous TaO_x_N_γ_ films, and TaN/TaO_x_ nano-multilayer films were fabricated via atomic layer deposition (ALD), and their microstructures as well as corrosion resistance on the surface of AISI 1045 steel were systematically investigated. The main conclusions were as follows: (1) Structural characteristics of the thin films. The ALD process enabled controllable growth of high-density, low-roughness (*R*q < 0.8 nm) Ta-based thin films. Specifically, TaN exhibited a polycrystalline face-centered cubic (fcc) structure, whereas the incorporation of oxygen or interfacial effects in multilayer structures could significantly suppress crystallinity, leading to an amorphous/nanocrystalline composite state. The homogeneous TaO_x_N_γ_ films adopted a fully amorphous solid solution structure due to the atomic-scale mutual solubility of oxygen and nitrogen. (2) Corrosion resistance ranking. In a 3.5 wt.% NaCl solution, the corrosion resistance followed the order: TaO_x_N_γ_ > TaN/TaO_x_ multilayer film > TaN. The homogeneous TaO_x_N_γ_ film demonstrated the best performance, with a charge transfer resistance (*R*_ct_ = 24.75 Ω·cm^2^) markedly higher than that of the substrate (*R*_ct_ = 5.94 Ω·cm^2^), and a corrosion current density (*I*_corr_ = 1.20 × 10^−6^ A/cm^2^) reduced to merely 7.45% of the substrate value. (3) Protective mechanism: The superior corrosion resistance of the amorphous TaO_x_N_γ_ film was attributed to the elimination of grain boundaries and pore defects, which maximized Cl^−^ diffusion blocking. In contrast, polycrystalline TaN with high conductivity accelerated corrosion. For multilayers, reducing sublayer thickness (5:5) enhanced interfacial scattering, while thicker sublayers (10:10) introduced residual stress and defects that weaken protection. In rapid screening salt spray tests (2 h exposure), the TaO_x_N_γ_ film exhibited no visible red rust originating from the steel substrate, whereas TaN and multilayer films showed early-stage corrosion within 10–60 min. This confirmed the superior initial barrier integrity of the TaO_x_N_γ_ film under accelerated corrosive attack. This study offers new insights into the design of highly corrosion-resistant atomic layer deposition (ALD) protective systems and broadened the application potential of tantalum-based materials in harsh corrosive environments.

## Figures and Tables

**Table 1:** *E*_corr_ and *I*_corr_ values obtained from potential polarization analysis on AISI 1045 substrate, TaN, homogeneous TaO_x_N_γ_, TaN/TaOx-5:5 and TaN/TaOx-10:10 mutilayered films in 3.5 wt.% NaCl solution.

	*E*_corr_ (V)	*I*_corr_ (A·cm^−2^)
AISI 1045	−0.823	1.61 × 10^−5^
TaN	−0.740	5.66 × 10^−6^
TaO_x_N_γ_	−0.754	1.20 × 10^−6^
TaN/TaO_x_-5:5	−0.665	2.94 × 10^−6^
TaN/TaO_x_-10:10	−0.706	2.89 × 10^−6^

^1^ The *E*_corr_ and *I*_corr_ values listed in the table represent the representative single results from three repeated measurements. ^2^ The ranking of corrosion resistance was primarily based on the *I*_corr_, which directly represents the corrosion rate.

**Table 2:** Relevant parameters of Nyquist curve fitting circuit diagram.

Samples	*L*/(H)	*R*_s_/Ω⋅cm^2^	*R*_ct_/Ω⋅cm^2^	*Q*_dl_/Ω^−1^cm^−2^Sn	n_dl_	*R*_f_/Ω⋅cm^2^	*Q*_f_/Ω^−1^cm^−2^⋅Sn	n_f_
AISI 1045	8.17 × 10^−8^	1.541	5.94	5.634 × 10^−6^	0.929	/	/	/
TaN	3.08 × 10^−8^	1.736	10.74	1.208 × 10^−6^	0.983	2.131	3.345 × 10^−4^	0.918
TaO_x_N_γ_	6.52 × 10^−8^	1.652	24.75	6.616 × 10^−7^	1	6.096	0.2839	1
TaN/TaO_x_-5:5	4.18 × 10^−8^	1.638	19.76	7.910 × 10^−7^	1	1.904	1.820 × 10^−4^	1
TaN/TaO_x_-10:10	6.54 × 10^−8^	1.662	18.17	7.753 × 10^−7^	1	1.601	1.359 × 10^−4^	1

^2^ Fitted parameters of Nyquist curve listed in the table represent the representative single results from three repeated measurements.

**Table 3:** Relevant parameters of Bode amplitude graph and Bode phase graph.

Samples	Bode Amplitude Plot Related Parameters		Bode Phase Diagram Related Parameters	
	0.1 Hz~3.16 kHz (log f: −1~3.5)		log f: 3~6 Hz	−Phase Peak (°)
	log|Z| (Ω·cm^2^)	|Z| (Ω·cm^2^)		
AISI 1045	0.9	8 × 10^4^	4.40	38
TaN	1.19	15.5 × 10^4^	4.55	49
TaO_x_N_γ_	1.41	25.7 × 10^4^	4.55	62
TaN/TaO_x_-5:5	1.39	24.5 × 10^4^	4.55	59
TaN/TaO_x_-10:10	1.35	22.9 × 10^4^	4.55	58

^3^ Relevant parameters of Bode amplitude and Bode phase graph listed in the table represent the representative single results from three repeated measurements.

## Data Availability

The data of this article are available from the corresponding author upon reasonable request.

## Supplementary Materials

The following supporting information can be downloaded at: https://www.mdpi.com/article/10.3390/nano16110688/s1, Table S1: Quantitative composition of TaO_x_N_γ_ thin films by XPS; Table S2: Comparison of ALD protective coatings for corrosion resistance.
