# Supplementary material for: Anti-Corrosion Properties of Tantalum-Based Composite Films Prepared by Atomic Layer Deposition
Source: Nanomaterials (Basel). 2026 Jun 1;16(11):688. doi: 10.3390/nano16110688 (PMC13258363; doi:10.3390/nano16110688)
Supplement: Supplementary file 1 [file nanomaterials-16-00688-s001.zip › nanomaterials-4232002-supplementary.pdf]

Table S1 Quantitative composition of TaO_xN_γ thin films by XPS

	Atomic %
Ta 4f	49.15
N 1s	18.72
O 1s	30.08
C 1s	2.05

Table S2 Comparison of ALD protective coatings for corrosion resistance

Reference	Technique	Material	Substrate	Medium	Key Results	Thickness
**This work**	**ALD**	**TaN, TaO_xN_γ, multilayers**	**AISI 1045 steel**	**3.5 wt.% NaCl**	**TaO_xN_γ: I_{corr}:1.20×10^{-6} A/cm^2, R_{ct}: 24.75 Ω·cm^2, 2 h salt spray: no red rust**	**~60 nm**
Shan et al[1]. 2008	ALD	TiO_2	Stainless steel	3 wt.% NaCl	E_{corr}: +0.33 V, I_{corr}: 6.3 × 10^{-8}A/cm^2 R_{ct}: 11.5× 10^5 Ω·cm^2	~60 nm
Díaz et al[2]. 2011	ALD	Al_2O_3	100Cr6 steel	0.2 M NaCl	Improved corrosion resistance	10–50 nm
Marin et al[3]. 2012	ALD	TiO_2/Al_2O_3	AISI 316L SS	0.1 M NaCl	Multilayer > single-layer	~50 nm
Díaz et al[4]. 2014	ALD	Al_2O_3/TaO_x	Carb on steel	Acidic NaCl	Higher stability than Al_2O_3	~50 nm
Hirpara et al[5]. 2021	Sputtering	TaON	SS 304	1 M NaCl	I_{corr}: 10^{-11} A/cm^2, R_p: 3.14 MΩ reduced the corrosion rate and enhanced the inhibition efficiency (more than 50%)	Not specified

[1] Shan C X, Hou X, Choy K-L. Corrosion resistance of TiO_2 films grown on stainless steel by atomic layer deposition. *Surface and Coatings Technology*, 2008, 202(11): 2399-402, https://doi.org/10.1016/j.surfcoat.2007.08.066.

[2] Potts S E, Schmalz L, Fenker M, et al. Ultra-Thin Aluminium Oxide Films Deposited by Plasma-Enhanced Atomic Layer Deposition for Corrosion Protection. *Journal of The Electrochemical Society*, 2011, 158(5): C132, https://doi.org/10.1149/1.3560197.

[3] Marin E, Guzman L, Lanzutti A, et al. Multilayer Al_2O_3/TiO_2 Atomic Layer Deposition coatings for the corrosion protection of stainless steel. *Thin Solid Films*, 2012, 522: 283-8.

[4] Harkonen E, Tervakangas S, Kolehmainen J, et al. Interface control of atomic layer deposited oxide coatings by filtered cathodic arc deposited sublayers for improved corrosion protection. *Materials Chemistry and Physics*, 2014, 147(3): 895-907, https://doi.org/10.1016/j.matchemphys.2014.06.035.

[5] Hirpara J, Chawla V, Chandra R. Anticorrosive Behavior Enhancement of Stainless Steel 304 through Tantalum-Based Coatings: Role of Coating Morphology. *Journal of Materials Engineering and Performance*, 2021, 30(3): 1895-905, https://doi.org/10.1007/s11665-021-05542-5.
